# Non-medical use of exogenous testosterone and anabolic–androgenic substances in young men: health, psychological, and fertility consequences

**DOI:** 10.3389/fendo.2026.1781416

**Published:** 2026-03-05

**Authors:** Krzysztof Kowalik, Patryk Harasny, Laura Kaliczyńska, Konrad Reweda-Kwiatkowski, Dariusz Starzyński, Michał Pawlak, Arkadiusz Waloryszak, Magdalena Ptak, Andrzej Modrzejewski, Dagmara Lisman

**Affiliations:** 1 Clinical Department of General Surgery, Pomeranian Medical University in Szczecin, Szczecin, Poland; 2 Department of Urology, Provincial Hospital, Poznań, Poland; 3 University of Szczecin, Szczecin, Poland; 4 Faculty of Health Sciences, Pomeranian Medical University in Szczecin, Szczecin, Poland; 5 Endocrinology Clinic, Sedimed Medical Centre, Szczecin, Poland; 6 Faculty of Dentistry, Pomeranian Medical University in Szczecin, Szczecin, Poland; 7 Independent Unit of Perineological Physiotherapy, Faculty of Health Sciences, Pomeranian Medical University in Szczecin, Szczecin, Poland; 8 Department of Genomics and Forensic Genetics, Pomeranian Medical University in Szczecin, Szczecin, Poland

**Keywords:** anabolic–androgenic steroids, endocrine disruption, hypogonadism, hypothalamic–pituitary–gonadal axis, male infertility, testosterone misuse, young men

## Abstract

The non-medical use of exogenous testosterone and other anabolic–androgenic steroids (AAS) has increased substantially in recent years, particularly among young men engaged in recreational strength training. Although often perceived as a means of enhancing muscle mass and physical performance, this practice represents a growing public-health concern due to its wide-ranging endocrine, reproductive, and multisystem adverse effects. This narrative review synthesizes current international evidence on the non-medical use of testosterone and AAS in non-professional athletic settings, with a primary focus on endocrine disruption and reproductive health. The review outlines the classification of commonly used anabolic–androgenic compounds, discusses their pharmacological mechanisms of action, and integrates clinical, experimental, and epidemiological data on associated adverse outcomes. Particular attention is given to suppression of the hypothalamic–pituitary–gonadal axis, impaired spermatogenesis, fertility disturbances, and the potential for long-term or persistent endocrine sequelae. In addition, psychological and behavioural factors contributing to AAS use—including muscle dysmorphia, social pressure, and body-image concerns—are discussed as important modulators of risk. The review also addresses current clinical approaches to the management of AAS-related complications, including strategies aimed at hormonal recovery and restoration of reproductive function. By presenting a comprehensive, mechanistic, and clinically oriented overview, this article highlights the need for increased awareness among clinicians and underscores priorities for future research and preventive interventions in endocrine and reproductive health.

## 1 Revised introduction

The non-medical use of exogenous testosterone among individuals engaged in strength-based sports has emerged as a widespread phenomenon, comparable in scale to other forms of substance misuse (1). In addition to athletes, men affected by muscle dysmorphia represent a particularly vulnerable group, as this disorder is associated with compulsive body image concerns and a heightened tendency toward performance-enhancing substance use (2). Although the exact prevalence of muscle dysmorphia remains uncertain, it is estimated to affect approximately 1–3% of young men, with population-based studies reporting rates of around 2–3% (2).

Available epidemiological data indicate that testosterone is frequently used in a manner inconsistent with medical recommendations. It is estimated that approximately 6% of men report non-medical testosterone use (3), while substantially higher prevalence rates have been observed among regular gym users (4). Considerable variability in reported figures may result from underreporting, social stigma, and reluctance to disclose substance use, as well as limited awareness of the pharmacological properties of agents being consumed.

Studies conducted among strength athletes and bodybuilders have consistently demonstrated high rates of anabolic–androgenic substance use. Reported prevalence varies across populations and study designs, with estimates ranging from approximately one-third to over one-half of respondents admitting to the use of performance-enhancing substances, including anabolic–androgenic steroids (5, 6). Discrepancies between studies may also stem from intentional concealment of substance use or misclassification, as some individuals mistakenly associate anabolic steroids with corticosteroids or common dietary supplements (7). Importantly, contamination of sports supplements with pharmacologically active substances, including anabolic–androgenic steroids and prohormones, has been repeatedly documented, representing a significant public health concern (7, 8).

Available data on the non-medical use of testosterone remain limited and heterogeneous across different regions worldwide. Evidence varies substantially between countries and populations, reflecting differences in study design, reporting practices, legal frameworks, and cultural attitudes toward anabolic–androgenic substance use. The first questionnaire-based study conducted in a Polish population demonstrated that testosterone use predominantly affects young adults, particularly men aged 18–40 years, with the highest prevalence observed among individuals engaged in regular strength training (9). A substantial proportion of users reported prolonged exposure to testosterone or other anabolic–androgenic substances, further emphasizing the potential risk of long-term adverse health consequences.

Despite the growing prevalence of anabolic–androgenic substance use, another critical issue is the level of awareness within the medical community. Data assessing physicians’ knowledge, attitudes, and clinical experience in managing patients who use exogenous testosterone are scarce in the Polish literature, limiting the ability to evaluate preparedness for addressing this emerging health problem. Evidence from international studies suggests that, although physicians may possess general theoretical knowledge regarding anabolic–androgenic steroids, their clinical awareness and experience remain insufficient (10). Importantly, patients may intentionally conceal testosterone use due to a lack of trust in medical personnel, further complicating diagnosis and management.

These observations underscore the urgent need for improved physician education and targeted clinical training focused on recognizing, monitoring, and managing the adverse effects associated with non-medical testosterone and anabolic–androgenic substance use. A comprehensive synthesis of current evidence is therefore essential to support clinical practice, inform preventive strategies, and guide future research in this rapidly evolving field.

## 2 Aim of the study

The aim of this review is to synthesize current evidence on the non-medical use of exogenous testosterone among young men engaged in recreational strength training and to discuss its associated health and psychological consequences. In this study, ‘young men’ refers to males aged 18–40 years, representing a population with stable endogenous androgen levels and minimal age-related hormonal variability.

Specifically, the review focuses on:

- the classification and key characteristics of the most commonly used anabolic–androgenic agents,
- their pharmacological effects and the most frequently reported adverse reactions,
- psychological determinants and consequences of anabolic–androgenic substance use, including muscle dysmorphia and social pressure,
- clinical management strategies, including approaches to restoring hypothalamic–pituitary–testicular (HPT) axis function in individuals abusing testosterone.

## 3 Research questions

### 3.1 Mental health and psychological consequences of testosterone misuse

To what extent is non-medical use of exogenous testosterone associated with mental health disturbances, including muscle dysmorphia, in young men engaged in recreational strength training?

### 3.2 Long-term HPT axis dysfunction and fertility impairment

What evidence exists regarding long-term disturbances of the hypothalamic–pituitary–testicular axis and fertility impairment following anabolic–androgenic substance abuse?

## 4 Prevalence, classification, and mechanisms of action of anabolic–androgenic substances

**Figure 1:**
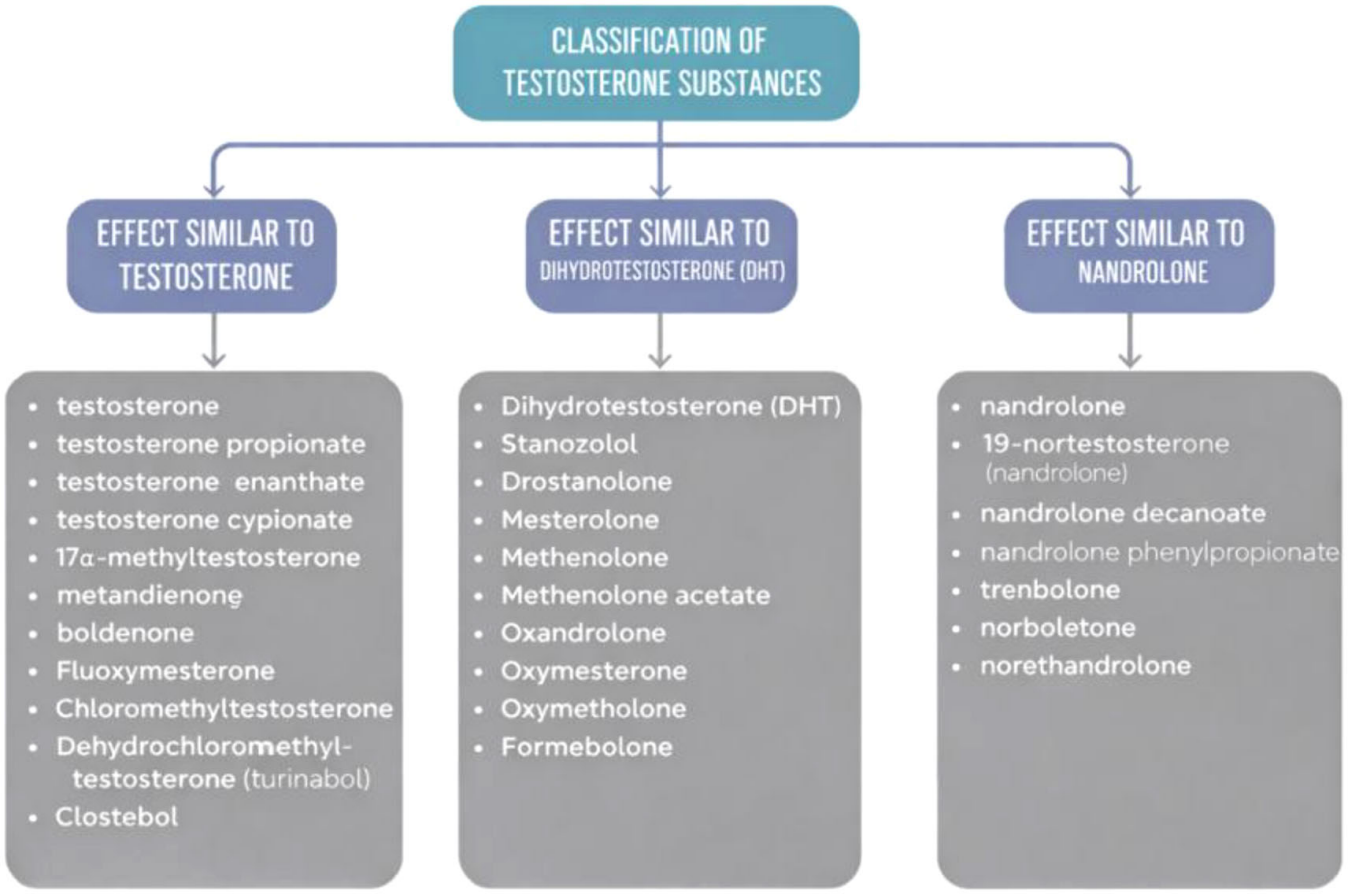
Diagram illustrating the classification of exogenous testosterone preparations with representative examples (11).

Although a uniform classification of anabolic–androgenic substances (AAS) has not been formally established, in practice a functional division based on chemical structure, pharmacokinetics, and route of administration is commonly applied, as illustrated in **Figure 1**.

The biological effects of testosterone are mediated both by direct binding and activation of the androgen receptor, as well as indirectly through its metabolic conversion to biologically active derivatives, including 5α-dihydrotestosterone (DHT) via 5α-reductase and oestradiol via aromatization, which subsequently act through androgen and oestrogen receptors (ERα/ERβ), respectively. The physiological response to exogenous testosterone is further modulated by individual genetic variability, particularly polymorphisms of the androgen receptor gene, which contribute to substantial interindividual differences in efficacy and adverse effect profiles (12).

The principal mechanisms of action of testosterone include:

- stimulation of protein synthesis and anabolic pathways,
- direct activation of androgen receptors in target tissues,
- psychological effects, such as alterations in mood, motivation, and libido.

Exogenous testosterone is available in multiple pharmaceutical formulations. The most commonly used preparations include intramuscular injections of oil-based esters, such as testosterone enanthate or cypionate. Alternative routes of administration include oral formulations, transdermal systems (gels and patches), and subcutaneous implants, which allow for more stable serum testosterone concentrations and individualized dosing strategies.

In therapeutic settings, testosterone doses typically range from approximately 50 to 250 mg per week. In contrast, doses used in non-medical contexts, particularly in sports doping, may be several times higher and have been reported to reach 500–5000 mg per week over extended cycles (13). Such regimens are often designed to maximize anabolic effects by combining multiple testosterone esters or synthetic analogues with differing durations of action. Prolonged exposure to supraphysiological doses may result in down-regulation of androgen receptor expression, potentially leading to diminished tissue responsiveness over time (13).

The most commonly used testosterone preparations and their derivatives are summarized in **Table 1**.

**Table 1:** The most commonly used exogenous testosterone preparations and their derivatives in strength-based doping (11, 13).

Substance	Route of administration	Typical therapeutic dose	Estimated non-medical (doping) dose	Typical dosing frequency	Remarks
Testosterone enanthate	IM	100–250 mg every 2–3 weeks	500–1500 mg/week	1–2×/week	Long-acting ester, slow release
Testosterone cypionate	IM	100–200 mg every 2 weeks	500–1200 mg/week	1×/week	Frequently used in strength-oriented doping
Testosterone propionate	IM	25–50 mg every 2–3 days	500–1000 mg/week	2–3×/week	Short-acting ester, rapid onset
Testosterone undecanoate	PO/SC	40–80 mg/day (PO)	200–400 mg/day (≈1400–2800 mg/week)	Daily	Lower oral bioavailability; depot IM formulations also available
Methenolone (enanthate/acetate)	IM/PO	~100 mg/week	400–800 mg/week	1–2×/week	Considered less androgenic; commonly used during cutting phases
Stanozolol	PO/IM	10–25 mg/day	50–100 mg/day (≈350–700 mg/week)	Daily	Frequently combined with testosterone
Nandrolone decanoate	IM	50–200 mg every 2–4 weeks	200–600 mg/week	1×/week	Synthetic derivative of 19-nortestosterone
Trenbolone (acetate/enanthate)	IM	No approved clinical use	200–700 mg/week	2–3×/week	Highly potent anabolic effects with numerous adverse effects

Legend, PO, *per os* (by mouth); SC, *sub cutem* (subcutaneous); IM, *intramuscularis* (intramuscular).

IM, intramuscular; PO, oral administration; SC, subcutaneous.

A review of the available literature indicates that anabolic–androgenic steroids are commonly administered using a strategy referred to as “stacking”, which involves the concurrent use of multiple substances and, in many cases, progressive dose escalation over cycles typically lasting 6–12 weeks. Such regimens have been reported to result in cumulative doses reaching 40–100 times higher than physiological testosterone levels. In some reports, recommended regimens for gym users differed only by the brand name of the preparation used during successive weeks, rather than by pharmacological rationale (14).

Another frequently described pattern of administration is “cycling”, which consists of alternating periods of steroid use and abstinence. During off-cycle phases, many users report the use of agents such as tamoxifen or human chorionic gonadotropin (hCG) in an attempt to stimulate endogenous testosterone production or restore testicular function. However, evidence supporting the effectiveness and safety of these practices remains limited and inconsistent in the scientific literature (15).

According to one cited study (16), the most commonly used anabolic–androgenic agents included testosterone enanthate and cypionate (96% of respondents), trenbolone (52%), drostanolone (39%), boldenone (38%), and nandrolone (33%). The estimated mean weekly androgen dose approached 1000 mg, with reported values ranging from 250 to 3300 mg per week. For comparison, standard testosterone replacement therapy for male hypogonadism typically does not exceed 100 mg per week. These findings highlight a substantial exceedance of physiological dosing levels among users, which is associated with a markedly increased risk of adverse health outcomes.

According to current evidence, no non-medical use of anabolic–androgenic steroids can be considered safe. The use of these substances for performance enhancement—particularly when administered in high doses or complex cycles—has been associated with a wide range of endocrine, metabolic, and cardiovascular disturbances, as well as psychological effects and the potential development of physical and psychological dependence (11, 17).

## 5 Reasons for the use of anabolic–androgenic steroids in amateur sports

### 5.1 Body image, masculinity, and social determinants

A muscular and well-defined physique constitutes an important component of identity and self-esteem for many men (18). In contemporary society, concerns related to physical appearance are commonly addressed through regular physical activity, dietary modification, and the use of nutritional supplements. However, when these strategies are perceived as insufficient to achieve desired body-image outcomes, some individuals seek faster and more effective methods, including the use of performance-enhancing substances such as anabolic–androgenic steroids (AAS).

In amateur sports, AAS use differs fundamentally from professional doping practices, as substances are typically consumed not to enhance competitive performance but to improve physical appearance and obtain social approval (19, 20). Evidence suggests that most AAS users are regular gym attendees (21), whose primary motivations include increasing muscle mass and strength, as well as enhancing overall physical attractiveness (22). Contributing factors include the widespread availability of AAS—particularly through online markets (23)—growing social normalization of their use, and increased emphasis on male body ideals promoted through social media platforms (24).

### 5.2 Role of gym culture and social media

Social interactions within gym environments may further reinforce AAS use by normalizing or encouraging such behaviours. Trainers, training partners, and peer groups often influence decision-making processes, either directly through recommendation or indirectly by fostering a permissive social climate toward substance use (25, 26). Concurrently, social media platforms amplify unrealistic body ideals, intensify appearance-related pressure, and increase exposure to highly muscular physiques portrayed by influencers and fitness models (27).

Although these platforms serve as a major source of information, they are also associated with the dissemination of misinformation (28) and have been linked to negative psychological outcomes, including body-image disturbances, disordered eating behaviours, and reduced well-being (29, 30). One proposed mechanism underlying these effects is frequent upward social comparison, whereby individuals evaluate their own appearance against idealized and often unattainable body standards (31, 32). Exposure to content promoting supplementation, intensive training, or a “bodybuilding lifestyle” may further shape nutritional and training behaviours among recreational exercisers (33), thereby exacerbating body dissatisfaction and increasing vulnerability to eating disorders (30, 34, 35).

### 5.3 Individual psychological and personality risk factors

At the individual level, negative body image represents a key risk factor for AAS use, particularly among young adult men with a strong desire to increase muscle mass (36). Prior to initiating AAS use, individuals more frequently exhibit low self-esteem, interpersonal difficulties, impulsivity, and a tendency toward risk-taking behaviours. A history of other substance use, higher socioeconomic status, or exposure to family violence or substance abuse has also been reported more often in this population (37, 38). Amateur bodybuilders commonly display symptoms of muscle dysmorphia and elevated impulsivity (39), while certain personality traits—such as neuroticism, narcissism, low agreeableness, and impaired delay of gratification—have been identified as predictors of AAS use (40, 41).

Importantly, no single factor independently determines the decision to use anabolic–androgenic steroids. Rather, AAS use in amateur sports results from the complex interaction of psychological, social, and environmental risk factors, which may act cumulatively or sequentially over time (42). Despite increasing awareness of the anabolic effects of these substances, numerous studies indicate that users often possess limited knowledge regarding their potential adverse health consequences (43–45). Even when risks are acknowledged, they are frequently perceived as insufficient to justify discontinuation. Consequently, targeted educational initiatives and improved access to qualified healthcare services may play a critical role in effective prevention and harm-reduction strategies (46, 47).

## 6 Selected psychological effects of anabolic–androgenic steroid use in amateur sports

### 6.1 Emotional and behavioural disturbances

The use of anabolic–androgenic steroids (AAS) in amateur sports has been associated with a wide range of emotional, cognitive, and behavioural effects. Commonly reported symptoms among users include increased irritability, impulsivity, mood instability, and a heightened tendency toward aggressive behaviour. In some individuals, more severe psychiatric manifestations—such as depressive disorders, manic episodes, or psychotic symptoms—have also been described (48–50).

### 6.2 Neurobiological and cognitive effects

Neurobiological research suggests that AAS exposure may affect central nervous system functioning by inducing both structural and functional alterations. These include changes within the cerebral cortex, reductions in grey matter volume, and increased amygdala volume (51–54). Such neuroanatomical and neurofunctional changes have been associated with impairments in emotional regulation and cognitive performance (55–58). Moreover, these alterations may contribute to increased anxiety, reduced self-control, and a greater propensity for impulsive or dominant behaviours, particularly in individuals with pre-existing traits such as poor impulse regulation or antisocial tendencies (59, 60).

### 6.3 Dependence, withdrawal, and suicidality

Evidence indicates that the risk of aggressive behaviour is further elevated when AAS are used concomitantly with alcohol or stimulant substances (61). Importantly, the behavioural effects of AAS appear to be modulated by individual personality characteristics, suggesting that not all users exhibit uniform psychological responses (59). Nevertheless, available evidence indicates that prolonged exposure to supraphysiological doses of AAS is associated with a substantial risk of adverse psychological outcomes, including the development of dependence and persistent emotional and interpersonal difficulties (62). It has been estimated that approximately one-third of AAS users may develop a dependence syndrome, characterized by withdrawal symptoms and continued use despite awareness of negative health or social consequences (62). In addition, an increased risk of severe depressive episodes and suicidal ideation has been reported during withdrawal phases (63, 64).

Overall, the literature highlights the heterogeneous and multifactorial nature of psychological responses to AAS use. The effects of these substances appear to be highly individual, influenced by biological predispositions, personality traits, and environmental factors. While some users report transient euphoria and increased self-confidence, others experience negative emotional states, including irritability, sadness, or heightened aggression (65–67).

Given the complexity of these interactions and the limited number of high-quality longitudinal studies, further research is warranted to better characterize the psychological consequences of anabolic–androgenic steroid use in amateur sports and to inform effective prevention and clinical management strategies.

## 7 Adverse effects of exogenous testosterone use

### 7.1 Cardiovascular toxicity

Chronic exposure to supraphysiological concentrations of anabolic–androgenic steroids (AAS) has been associated with systemic toxicity affecting cardiovascular tissues. Evidence derived from human observational studies, autopsy findings, and experimental models suggests that sustained androgen receptor overstimulation may induce structural, functional, and metabolic alterations that compromise cardiovascular homeostasis (68, 69). Proposed mechanisms include oxidative stress, mitochondrial dysfunction, dyslipidaemia, and genomic modulation of hypertrophic and fibrotic signalling pathways (70).

Cardiovascular pathology represents one of the most clinically relevant consequences of AAS misuse. Observational studies in athletic populations have reported persistent systolic hypertension, concentric left ventricular hypertrophy, and impaired diastolic function during and following anabolic steroid exposure (71). Histopathological analyses frequently demonstrate myocardial fibrosis, vascular inflammation, and focal necrosis, indicating maladaptive cardiac remodelling (72). Echocardiographic and cardiac magnetic-resonance imaging studies have further documented reduced ventricular compliance and prolonged relaxation times, which appear to correlate with cumulative duration of AAS exposure. At the molecular level, excessive androgen receptor activation has been shown to stimulate phosphoinositide-3-kinase (PI3K)/Akt and mitogen-activated protein kinase (MAPK) signalling pathways, promoting cardiomyocyte hypertrophy while simultaneously enhancing mitochondrial oxidative injury (73).

Endothelial dysfunction is considered a key link between AAS abuse and macrovascular disease. Supraphysiological testosterone concentrations have been shown to reduce endothelial nitric oxide synthase expression and nitric oxide bioavailability, thereby favouring vasoconstriction and increased arterial stiffness (73). These effects coexist with characteristic lipid profile alterations, including elevated low-density lipoprotein cholesterol, reduced high-density lipoprotein cholesterol, and increased triglyceride levels, which together may accelerate atherogenic processes (70). Prothrombotic changes, such as erythrocytosis and enhanced platelet reactivity, further contribute to an increased risk of acute cardiovascular events, including myocardial infarction and sudden cardiac death. Collectively, these abnormalities resemble a cardiometabolic risk profile that may persist even after cessation of AAS use (72).

Oxidative stress appears to represent a central mechanistic pathway linking molecular signalling alterations with structural cardiac pathology. Experimental studies indicate that supraphysiological testosterone exposure increases reactive oxygen species generation within cardiomyocytes while reducing the activity of endogenous antioxidant enzymes, including superoxide dismutase and glutathione peroxidase (72). This redox imbalance promotes mitochondrial DNA damage, lipid peroxidation, and activation of apoptotic pathways, ultimately contributing to myofibrillar disorganisation and interstitial fibrosis (73).

Electrophysiological disturbances may arise as a consequence of these structural and biochemical changes. AAS exposure has been associated with prolongation of ventricular repolarisation, increased dispersion of action potential duration, and reduced heart-rate variability, all of which facilitate arrhythmogenesis (71). Case series of sudden cardiac death in young athletes frequently report myocardial hypertrophy and fibrosis, supporting the presence of an arrhythmogenic substrate in long-term AAS users (72).

### 7.2 Hepatic toxicity

Hepatic dysfunction represents another major adverse effect associated with chronic exposure to supraphysiological androgen levels. As the primary organ responsible for steroid metabolism, the liver is particularly susceptible to the toxic effects of 17-α-alkylated oral anabolic–androgenic steroids, such as stanozolol and methyltestosterone (74). Clinical manifestations range from asymptomatic elevations in serum transaminases to more severe conditions, including cholestatic jaundice, peliosis hepatis, hepatic adenomas, and, in rare cases, hepatocellular carcinoma (75). Histological findings from both animal models and human studies have described sinusoidal dilatation, microvesicular steatosis, and pericentral necrosis, changes commonly associated with oxidative and inflammatory stress. The severity of hepatic injury appears to correlate with both cumulative dose and duration of exposure (74).

Mechanistic studies suggest that anabolic–androgenic steroids impair mitochondrial β-oxidation and deplete intracellular glutathione stores, resulting in lipid peroxidation and activation of apoptotic signalling pathways. Experimental models demonstrate that high-dose testosterone administration increases hepatic reactive oxygen species production while suppressing endogenous antioxidant enzyme activity (74). These biochemical disturbances are accompanied by mitochondrial swelling and endoplasmic-reticulum stress, ultimately leading to hepatocyte injury and cell death. In addition, excessive androgen receptor activation alters transcriptional regulation of genes involved in glucose and lipid metabolism, thereby promoting hepatic steatosis and insulin resistance (72). Such metabolic reprogramming may contribute to both hepatic and systemic complications (74).

Intrahepatic cholestasis observed in anabolic steroid users has been linked to impaired bile-acid transport. Androgens have been shown to downregulate the expression of bile-salt export pump (BSEP) and multidrug resistance–associated proteins (MRPs), resulting in bile-acid retention and cytotoxic accumulation within hepatocytes. Histopathological examinations often reveal canalicular bile plugs and periportal inflammation, supporting a direct toxic mechanism. These findings indicate that hepatic toxicity associated with anabolic–androgenic steroid use can be severe and, in some cases, life-threatening (75).

The metabolic consequences of hepatic androgen exposure extend beyond the liver itself. Prolonged anabolic steroid use has been associated with impaired systemic insulin signalling, elevated fasting glucose levels, and dyslipidaemia (70). This adverse metabolic profile may further exacerbate cardiovascular risk by increasing oxidative stress and endothelial dysfunction, as androgen-mediated activation of metabolic pathways alters myocardial energy balance and redox homeostasis (73). Comparative analyses between physiological testosterone replacement therapy and supraphysiological anabolic steroid abuse underscore the importance of dose and molecular structure in determining toxicity. Testosterone replacement in hypogonadal men has been associated with favourable metabolic effects, including improved lipid profiles and glycaemic control (72). In contrast, synthetic anabolic–androgenic derivatives used in non-medical settings are chemically modified to resist hepatic metabolism, thereby prolonging androgen receptor activation and increasing the risk of hepatotoxic accumulation (74).

### 7.3 Endocrine and reproductive consequences

Importantly, recovery of endocrine function following cessation of AAS use is highly dependent on the duration, dose, and pattern of exposure. While short-term or single-cycle AAS use is often followed by partial or complete restoration of hypothalamic–pituitary–gonadal (HPG) axis function, prolonged or repeated exposure to supraphysiological doses is associated with an increased risk of incomplete or delayed recovery.

Prolonged exposure to supraphysiological doses of exogenous testosterone or synthetic anabolic–androgenic steroids (AAS) is associated with marked suppression of the hypothalamic–pituitary–gonadal (HPG) axis through negative feedback mechanisms that inhibit gonadotropin secretion (76). Elevated androgen and estradiol concentrations reduce gonadotropin-releasing hormone (GnRH) pulsatility at the hypothalamic level and suppress luteinising hormone (LH) and follicle-stimulating hormone (FSH) release from the anterior pituitary. These changes result in reduced intratesticular testosterone concentrations, testicular atrophy, and impaired spermatogenesis (77).

Importantly, cessation of AAS use does not invariably lead to full recovery of endocrine function. Observational studies indicate that subnormal serum testosterone levels and clinical features of androgen deficiency may persist for months or even years following withdrawal (77). Former users commonly report erectile dysfunction, fatigue, depressive symptoms, and loss of muscle mass (78). This persistent state, referred to as anabolic-steroid–induced hypogonadism (ASIH), has been attributed to prolonged suppression of hypothalamic GnRH signalling, desensitisation of pituitary gonadotropes, and potential structural or functional impairment of Leydig cells (79).

Histopathological evidence supports these proposed mechanisms. Testicular biopsies obtained from long-term AAS users have demonstrated depletion of germ cells and vacuolisation of Sertoli cells (68). In addition, interstitial fibrosis accompanied by reduced Leydig-cell density has been reported, suggesting chronic remodelling of testicular tissue. Similar alterations have been reproduced in animal models exposed to testosterone esters or nandrolone decanoate, in which mitochondrial dysfunction and caspase-mediated apoptosis occur within Leydig and Sertoli cells (79).

Emerging epigenetic evidence—derived predominantly from experimental animal models—suggests that prolonged or repeated exposure to supraphysiological doses of anabolic–androgenic steroids may induce persistent molecular alterations in the male reproductive system. Studies in rodents exposed to testosterone esters or nandrolone derivatives for several weeks to months have demonstrated changes in DNA methylation patterns and histone modifications affecting genes involved in spermatogenesis, germ-cell differentiation, and chromatin remodelling. Importantly, these epigenetic alterations have been shown to persist beyond the period of active androgen exposure, even after partial normalisation of circulating hormone levels. Animal studies further indicate that chronic paternal exposure to anabolic–androgenic steroids prior to conception may alter sperm-borne epigenetic signatures and adversely affect fertility-related outcomes in offspring, although direct evidence in humans remains limited and requires further investigation (80).

Several therapeutic strategies have been explored to promote endocrine and reproductive recovery following AAS cessation. Pharmacological stimulation of the HPG axis using human chorionic gonadotropin (hCG) and selective oestrogen-receptor modulators (SERMs) has been shown to restore endogenous testosterone production and spermatogenesis in a substantial proportion of affected individuals (77). In cases of prolonged FSH suppression, FSH analogues may be considered to support germ-cell maturation (81). Such interventions require careful clinical supervision, as inappropriate use may precipitate gynecomastia or further hormonal imbalance (80). Adjunctive approaches aimed at reducing oxidative stress have also been investigated. Antioxidant supplementation with agents such as coenzyme Q10, L-carnitine, and vitamin E has been associated with improvements in sperm motility and reductions in sperm DNA fragmentation in experimental models of androgen-induced infertility (79). In refractory cases characterised by persistent azoospermia, assisted reproductive techniques—including testicular sperm extraction (TESE) and intracytoplasmic sperm injection (ICSI)—may enable conception, although success depends on residual spermatogenic capacity (80).

### 7.4 Multisystem effects

Beyond the cardiovascular and hepatic axes, chronic exposure to anabolic–androgenic steroids (AAS) has been associated with adverse effects across multiple additional organ systems. The kidneys appear to be frequent, though often under-recognised, targets of anabolic-steroid toxicity. Long-term AAS use has been linked to glomerular hypertrophy and focal segmental glomerulosclerosis, likely secondary to hyperfiltration, systemic hypertension, and dyslipidaemia. These alterations may be further exacerbated by oxidative stress and activation of profibrotic cytokines, such as transforming growth factor β, ultimately contributing to the development of chronic kidney disease (82).

The musculoskeletal system—paradoxically the primary target of anabolic effects—is also vulnerable to injury. Rapid muscle hypertrophy without proportional adaptation of tendinous structures predisposes users to tendon rupture, particularly involving the Achilles and biceps tendons. In adolescent users, premature epiphyseal closure has been reported and may result in impaired longitudinal growth. Histological studies demonstrate disorganised collagen fibres and increased matrix metalloproteinase activity within tendons, changes associated with oxidative stress and disrupted extracellular-matrix turnover (83).

Cutaneous and adnexal manifestations are among the most commonly reported effects of AAS use. Excess androgen activity promotes sebaceous gland hypertrophy and increased sebum production, leading to acne, folliculitis, and androgenic alopecia. Although these dermatological changes are not life-threatening, they frequently contribute to significant psychosocial distress and have been associated with systemic inflammatory activation (70).

Alterations in immune function have also been described. Experimental data suggest that AAS exposure may suppress lymphocyte proliferation and reduce immunoglobulin production, indicating a degree of immunomodulation. Clinically, users may exhibit increased susceptibility to bacterial skin infections and respiratory tract infections, particularly when injectable preparations are administered under non-sterile conditions (84). The interaction between oxidative stress and impaired immune surveillance may further amplify systemic inflammation observed in hepatic and vascular tissues (70).

Respiratory and haematological systems may be indirectly affected through androgen-induced erythropoiesis and fluid retention. Increased erythropoietin levels and red blood cell mass can result in secondary polycythaemia, elevated blood viscosity, and heightened thromboembolic risk, consistent with the pro-coagulant state observed in cardiovascular studies (73). In addition, testosterone administration has been associated with worsening of sleep-disordered breathing and obstructive sleep apnoea in susceptible individuals, thereby compounding cardiovascular strain and arrhythmic risk (85).

Emerging evidence also implicates the gastrointestinal tract as a potential target of steroid toxicity. Chronic AAS exposure has been shown to alter gut microbiota composition, increase intestinal permeability, and enhance systemic oxidative stress. These changes may impair nutrient absorption and promote hepatic inflammation via the gut–liver axis (85, 86).

Collectively, these multisystem observations support the concept that anabolic–androgenic steroid toxicity represents a diffuse endocrine–metabolic disorder rather than isolated organ-specific pathology. The recurring interplay of oxidative stress, inflammatory activation, and disrupted cellular signalling provides a unifying mechanistic framework linking renal, musculoskeletal, cutaneous, immunological, respiratory, gastrointestinal, and reproductive dysfunctions to those already described in cardiovascular, hepatic, and gonadal tissues (83).

Taken together, the current body of evidence suggests that supraphysiological androgen exposure disrupts endocrine regulation at multiple levels—from hypothalamic control to testicular cellular architecture—through oxidative, mitochondrial, and epigenetic mechanisms (80). The resulting hypogonadism and impairment of fertility may persist in a subset of individuals even after cessation of anabolic–androgenic steroid use, raising concern regarding long-term or potentially irreversible remodelling of the reproductive axis (77). Pharmacological stimulation of gonadotropin secretion, antioxidant-based interventions, and assisted reproductive techniques may enable partial recovery in selected cases; however, prevention and early intervention remain the most effective strategies for limiting long-term endocrine and reproductive harm (76).

To facilitate synthesis of the extensive narrative data presented in Sections 6 and 7, a summary table is provided below, integrating the major psychological and physical adverse effects associated with non-medical testosterone and anabolic–androgenic steroid use across organ systems (**Table 2**).

**Table 2:** Summary of major psychological and physical adverse effects associated with non-medical testosterone and anabolic–androgenic steroid use.

System/domain	Key adverse effects	Proposed mechanisms	Sections
Psychological/Behavioural	Irritability, impulsivity, aggression, mood instability, anxiety, depressive symptoms	Androgen receptor overstimulation in limbic regions; altered serotonergic and dopaminergic signalling	6.1
Neurocognitive	Cognitive impairment, emotional dysregulation, reduced impulse control	Structural and functional brain changes (grey matter reduction, amygdala enlargement); neurotoxicity	6.2
Dependence & Withdrawal	Dependence syndrome, withdrawal symptoms, suicidality	Neuroadaptation to supraphysiological androgen exposure; dysregulation of reward pathways	6.3
Cardiovascular	Hypertension, cardiomyopathy, arrhythmias, sudden cardiac death	Oxidative stress, myocardial fibrosis, dyslipidaemia, endothelial dysfunction	7.1
Hepatic	Cholestasis, peliosis hepatis, hepatic adenomas, liver injury	Impaired mitochondrial β-oxidation, oxidative stress, altered bile-acid transport	7.2
Endocrine/Reproductive	Hypogonadism, infertility, testicular atrophy, impaired spermatogenesis	HPG-axis suppression, Leydig/Sertoli cell dysfunction, epigenetic alterations	7.3
Renal	Glomerular hypertrophy, focal segmental glomerulosclerosis	Hyperfiltration, hypertension, profibrotic cytokine activation	7.4
Musculoskeletal	Tendon rupture, impaired growth (adolescents)	Disproportionate muscle hypertrophy, collagen disorganization	7.4
Cutaneous	Acne, folliculitis, androgenic alopecia	Sebaceous gland hyperplasia, androgen excess	7.4
Immune/Hematologic	Infections, polycythaemia, thromboembolic risk	Immunomodulation, erythropoiesis, increased blood viscosity	7.4
Gastrointestinal/Metabolic	Dysbiosis, insulin resistance, metabolic dysregulation	Gut–liver axis disruption, oxidative stress	7.4

## 8 Discussion

In recent years, a substantial increase in testosterone use among men has been observed. This phenomenon appears to be multifactorial, driven by increased awareness of androgen deficiency syndromes, the expanding off-label prescription of testosterone preparations, and the widespread non-medical use of anabolic–androgenic steroids (AAS) to enhance physical performance and appearance. While this issue affects professional athletes, it is particularly prominent among individuals engaged in recreational strength and physique-oriented sports, where the primary motivation is often aesthetic rather than competitive (9).

A particularly vulnerable subgroup includes individuals with muscle dysmorphia (MD), a condition characterised by a persistent belief that one’s body is insufficiently muscular despite objectively normal or highly developed musculature. This disorder is closely linked to culturally reinforced masculine ideals, especially in Western societies, where muscularity is associated with strength, dominance, and masculinity. Previous studies and reviews have consistently reported an association between muscle dysmorphia and non-medical AAS use (87). Quantitative studies further indicate that individuals with MD who use anabolic steroids exhibit more severe body-image disturbances, more extreme exercise and dietary behaviours, and higher levels of psychopathology compared with non-using counterparts (87). The appeal of AAS in this population is largely attributable to the promise of rapid and pronounced increases in muscle mass.

Reliable data on the true prevalence of illegal testosterone use remain limited. Most epidemiological studies assess anabolic steroid or hormone use as a broad category rather than focusing on testosterone specifically, likely reflecting the common practice of combining multiple agents within androgen cycles. In addition, users frequently co-administer other substances intended to enhance performance or physique, including growth hormone, thyroid hormones, and insulin, further complicating prevalence estimates and risk assessment (16, 88).

A large meta-analysis by Sagoe et al., encompassing 271 studies, estimated the average lifetime prevalence of androgen use to be 6.4% among men and 1.6% among women. Notably higher prevalence rates were reported among recreational athletes (18.4%), competitive athletes (13.4%), prison inmates (12.4%), drug users (8.0%), and high school students (2.3%), compared with non-athletes (1.0%) (38). These findings underscore the broad penetration of androgen use beyond elite sports settings.

Comparable trends have been reported in regional studies. Montuori et al. conducted a cross-sectional survey of 107 bodybuilders recruited from gyms in the Naples metropolitan area and found that 35.5% admitted to the illegal use of hormones, although specific substances were not identified (89). Similarly, a Polish questionnaire-based study involving 179 gym users in Wrocław reported that 35.2% of respondents had used testosterone or other anabolic–androgenic substances (9). The primary motivations included improvement of training performance, physique enhancement, and increased resistance to physical exertion. Peer influence, internet sources, publications, and personal trainers were the most commonly cited motivators, while the majority of users reported no medical consultation prior to substance use.

The reviewed literature consistently demonstrates that anabolic–androgenic steroid use is associated with a wide spectrum of adverse effects, encompassing cardiovascular, neuropsychiatric, hepatic, endocrine, and reproductive systems. Cardiovascular complications, including cardiomyopathy and structural cardiac remodelling, appear to represent the most clinically significant outcomes, as comprehensively described by Iliakis and Stamou (90). In parallel, accumulating evidence suggests that AAS exposure may exert neurotoxic effects, contribute to cognitive impairment, and induce long-lasting behavioural and affective disturbances (91).

Hepatic toxicity and endocrine disruption further compound the systemic burden of AAS misuse. Suppression of the hypothalamic–pituitary–gonadal axis, impaired spermatogenesis, and reduced fertility represent particularly concerning consequences, with potential implications for the rising prevalence of infertility among young couples. Importantly, these effects may persist long after cessation of steroid use, as highlighted in both clinical and experimental studies.

An additional challenge in assessing the safety profile of testosterone and anabolic–androgenic steroid misuse is the high prevalence of polypharmacotherapy among non-medical users, which substantially confounds attribution of adverse effects to any single agent (92). In practice, AAS are frequently used in “stacks” combining multiple injectable and oral compounds, often alongside ancillary drugs intended to enhance physique outcomes or mitigate side effects. Commonly co-administered agents include aromatase inhibitors (e.g., anastrozole or letrozole) to reduce estrogenic effects, selective oestrogen receptor modulators (SERMs; e.g., tamoxifen or clomiphene) and/or human chorionic gonadotropin (hCG) as part of post-cycle therapy or to counteract hypogonadism, as well as 5α-reductase inhibitors for androgenic hair and skin effects. In addition, diuretics are frequently used during “cutting” or contest preparation to reduce water retention, increasing the risk of dehydration, electrolyte disturbances, arrhythmias, and acute kidney injury (92, 95).

Stimulants and sympathomimetics (including high-dose caffeine-containing preparations and other weight-loss agents), thyroid hormones, growth hormone, insulin and insulin-like growth factor-1–related strategies, and selective androgen receptor modulators (SARMs) are also reported, further increasing cardiometabolic and endocrine risk through complex pharmacodynamic and pharmacokinetic interactions (93, 94).

Importantly, such multi-drug regimens may contribute to several of the adverse outcomes attributed to AAS in observational studies. For example, hepatotoxicity may be amplified not only by 17α-alkylated oral steroids but also by concomitant use of other hepatically metabolized agents, alcohol, contaminated or counterfeit products, and high-dose supplements (93, 95). Similarly, thromboembolic events and cardiovascular toxicity may reflect the combined effects of erythrocytosis, dehydration induced by diuretics, stimulant-related vasoconstriction, and adverse lipid alterations (92, 95). Therefore, careful consideration of polypharmacy is essential when interpreting clinical reports and epidemiological data on testosterone and AAS-related harm.

## 9 Summary

The available literature indicates that non-medical use of exogenous testosterone and other anabolic–androgenic steroids (AAS) is associated with a broad spectrum of multisystem adverse effects mediated by oxidative stress, mitochondrial dysfunction, endocrine disruption, and epigenetic mechanisms. Cardiovascular complications—including myocardial hypertrophy, arrhythmias, endothelial dysfunction, and accelerated atherosclerotic processes—appear to represent the most clinically significant outcomes and may persist in some individuals even after cessation of steroid use. Hepatic injury, ranging from reversible cholestatic changes to severe conditions such as peliosis hepatis, constitutes another major risk, particularly among users of 17-α-alkylated oral compounds.

Endocrine suppression remains a characteristic feature of prolonged AAS exposure. Persistent hypogonadism, testicular atrophy, and impaired spermatogenesis have been frequently reported, and complete recovery of reproductive function is not invariably achieved. Histopathological and experimental findings suggest that structural gonadal alterations and epigenetic changes may contribute to prolonged fertility impairment. Although pharmacological stimulation of the hypothalamic–pituitary–gonadal axis using agents such as human chorionic gonadotropin and selective oestrogen-receptor modulators may promote recovery, these interventions require specialised medical supervision.

In addition to organ-specific toxicity, AAS use has been associated with neuropsychiatric consequences, including mood instability, aggression, depressive symptoms, and the development of dependence syndromes. These effects may impair individual functioning and contribute to broader public-health concerns, including increased engagement in risky behaviours and an elevated mental-health burden.

Overall, the evidence supports the view that anabolic–androgenic steroid misuse represents a complex endocrine–metabolic and public-health issue rather than an isolated doping-related practice.

## 10 Limitations of the study

This study is a narrative review, which entails several important limitations. First, available data on the non-medical use of testosterone and other anabolic–androgenic steroids (AAS) remain fragmentary and are frequently based on self-reported declarations by users. Given the illegal nature of AAS use in many settings and the ethical impossibility of conducting long-term interventional studies with supraphysiological androgen doses in humans, a substantial proportion of cases likely remain unreported, and the true prevalence of this phenomenon may be underestimated in the existing literature.

Second, research in this field is highly heterogeneous and predominantly observational in nature. Ethical constraints preclude controlled long-term AAS administration studies in humans; consequently, much of the available evidence derives from observational studies, case series, and case reports. As a result, critical information regarding the exact compounds used, dosages, duration of exposure, cycling patterns, and the concurrent use of additional substances in multi-drug regimens—whether intentional or unintentional—often relies on retrospective self-reporting by users, which is inherently subject to recall bias and misclassification.

Third, many studies address anabolic–androgenic steroids as a broad category without distinguishing testosterone as an individual compound. This lack of specificity complicates precise evaluation of the pharmacological and toxicological effects attributable to particular preparations. In addition, complex non-medical regimens—often involving the concurrent use of multiple agents such as thyroid hormones, growth hormone, insulin analogues, stimulants, or other performance- and physique-enhancing substances—further hinder interpretation of reported outcomes.

Importantly, controlled human data are largely limited to short-term experimental studies. A notable example is the placebo-controlled trial by Bhasin et al., which demonstrated dose-dependent anabolic effects of supraphysiological testosterone enanthate administered for 10 weeks in healthy men, providing valuable mechanistic insight under rigorously controlled conditions but without addressing long-term safety outcomes (96).

Finally, most published studies focus predominantly on young men attending gyms, which limits the generalisability of findings to other populations, including women, adolescents, and individuals with differing health profiles or patterns of substance use.

Despite these limitations, the available evidence suggests that non-medical AAS use is associated with substantial multi-organ health risks, underscoring the need for well-designed prospective observational studies, improved surveillance of non-medical androgen use, and the development of targeted preventive and educational programmes for high-risk groups.

The dosage ranges reported in this review are derived predominantly from observational evidence, including cross-sectional and questionnaire-based studies as well as retrospective self-reports from non-medical users, supplemented by case series describing adverse outcomes associated with supraphysiological androgen exposure.

## 11 Conclusions

1. The growing body of medical evidence supports the association between non-medical use of anabolic–androgenic steroids (AAS) and substantial health risks. Accurate assessment of the scope of this phenomenon remains challenging due to its predominantly clandestine nature, frequent polypharmacy, and the widespread availability of unregulated substances of uncertain composition and purity. Nevertheless, available data suggest a substantial increase in AAS use, particularly among young men in their reproductive years.
2. From a clinical perspective, exposure to anabolic–androgenic steroids should be considered in the differential diagnosis of a wide range of conditions affecting physically active young adults, including cardiomyopathy, arrhythmias, infertility, hepatic dysfunction, mood instability, and unexplained endocrine abnormalities. Given the potential for long-term and, in some cases, persistent complications, early identification and appropriate medical evaluation are of critical importance.
3. From a public-health standpoint, the rising prevalence of AAS use appears to be driven by the normalisation of muscular body ideals, the influence of social media, peer reinforcement within gym environments, and the ease of online access to illicit hormonal agents. Addressing these determinants requires targeted interventions. Educational initiatives, enhanced physician training, and the availability of accessible, non-judgmental healthcare services may help reduce harm and encourage affected individuals to seek professional support.
4. Ultimately, prevention remains the most effective strategy. Increasing awareness of the documented medical risks associated with anabolic–androgenic steroid misuse—rather than focusing exclusively on athletic or aesthetic motivations—may help limit the progression of this growing public-health challenge.
